# A cross-sectional study about associations between personality characteristics and mental health service utilization in a Korean national community sample of adults with psychiatric disorders

**DOI:** 10.1186/s12888-017-1322-2

**Published:** 2017-05-05

**Authors:** Subin Park, Yeeun Lee, Su Jeong Seong, Sung Man Chang, Jun Young Lee, Bong Jin Hahm, Jin Pyo Hong

**Affiliations:** 1 Department of Research Planning, Mental Health Research Institute, National Center for Mental Health, Seoul, South Korea; 2 Department of Psychology, Korea University, Seoul, South Korea; 3 Department of Psychiatry, Hallym University Medical Center, Seoul, South Korea; 4 Department of Psychiatry, Kyungpook National University School of Medicine, Daegu, South Korea; 5 Department of Psychiatry and Behavioral Science, Seoul National University College of Medicine, Seoul Metropolitan Government-Seoul National University Boramae Medical Center, Seoul, South Korea; 6 Department of Psychiatry and Behavioral Science, College of Medicine, Seoul National University, Seoul, South Korea; 7 Department of Neuropsychiatry, Seoul National University Hospital, Seoul, South Korea; 8 Department of Psychiatry, Samsung Medical Center, Sungkyunkwan University School of Medicine, 81 Irwon-ro Gangnam-gu, Seoul, 06351 Republic of Korea

**Keywords:** Mental health service utilization, Personality traits, Lifetime psychiatric diagnoses, KECA

## Abstract

**Background:**

Personality traits are not only associated with psychiatric symptoms, but also with treatment seeking behavior. Our purpose was to examine the relationship between mental health service utilization and personality characteristics in a nationwide community sample of Korean adults.

**Method:**

Of the 6022 subjects aged 18–74 years who participated in the Korean Epidemiologic Catchment Area study, 1544 (25.6%) with a lifetime diagnosis of any DSM-IV psychiatric disorder were analyzed. Diagnostic assessments were based on the Composite International Diagnostic Interview and personality constructs were measured by Big Five Personality Inventory-10.

**Results:**

Of the 1544 participants, 275 (17.8%) had used mental health services. Multivariate analyses revealed positive associations between mental health service utilization and both neuroticism and openness, and an inverse association between mental health service utilization and agreeableness.

**Conclusions:**

These findings suggest that specific personality traits may have a role in treatment-seeking behaviors for mental health problems independent of the psychiatric disorder.

## Background

It is paramount for an individual with a mental difficulty to obtain an appropriate intervention. Although the proportion of treatment recipients is increasing, the majority of patients with psychiatric disorders worldwide still do not receive any professional health care [1, 2]. Wang et al. [2] reported the percentage of 12-month mental health service use across 17 countries; it ranged from 11.0% in China to 59.7% in United States for severe mental disorders, and 1.7% to 26.2%, respectively, for mild mental disorders. The unmet needs for mental health service would appear to be more serious in developing countries [2–4].

With the goal of improving access to mental health care, epidemiological studies have identified factors associated with mental health service utilization (MHSU) (see [5]. Most studies focused on socio-demographical predisposing factors, encompassing ethnic minorities [6, 7], male gender [8], and those with low education level [9]. Cultural barriers, such as stigma [10], misconception of mental health problems [11], and mental health illiteracy [12] may also shape negative attitudes toward professional service.

In line with efforts to investigate barriers to those seeking help for mental illness, Goodwin et al. [9] underscored the effect of personality traits on MHSU. Personality factors are not just associated with mental illness itself [13], but may also affect treatment seeking behavior. McWilliams et al. [14] stressed considering personality-related factors to expand the scope of factors that affect MHSU, documenting the positive association of outpatient MHSU with self-criticism and locus of control. Most studies on personality traits and MHSU have examined the associations with particular personality traits. Using a nationally representative sample of Americans, Goodwin et al. [9] revealed that five factor model was closely related to mental health service use among individuals with and without a diagnosed psychiatric disorder. Neuroticism was associated with increased MHSU, while conscientiousness was related to decreased MHSU among patients with any mental disorder. Ten Have et al. [15] also confirmed that neuroticism was related to increased access to the mental health sector, regardless of whether individuals had an emotional disorder. In addition, Schomerus et al. [16] suggested that high conscientiousness was related to higher access to mental health care among depressive patients, and Scholte-Stalenhoef et al. [17] reported a positive association between openness and ambulatory care use among those with early psychotic symptoms. Considered together, the data indicate that MHSU is clearly associated with higher levels of neuroticism. But, the associations with the other four factors are mixed or unclear.

A negative attitude towards mental illness is pervasive in Asian culture [10, 18–20]. Asians also seem to minimize mental health problems, considering them as problem to endure and overcome by their own will [21, 22]. Asians who tend to suppress their emotions are less likely to recognize or admit their mental health needs [11]. This cultural attitude fuels reluctance regarding MHSU [11, 18, 23]. Considering the identified cultural barrier for people from Asians cultures, the role of personality traits in MHSU might differ among Asians. The present study used a nationally representative sample of Koreans to investigate the relationship between personality traits and MHSU among Korean adults with diagnosed mental disorders.

## Methods

### Subjects

Analyzed data were from the Korean Epidemiologic Catchment Area (KECA) studies of 2001 [24] and 2006 [25]. KECA used the Korean version of the Composite International Diagnostic Interview 2.1 (K-CIDI 2.1) [26]. KECA also included a 2011 follow-up and examined lifetime and 1-year prevalence, socioeconomic status, and co-morbidities of mental illness in the Diagnostic and Statistical Manual of Mental Disorders, Fourth Edition (DSM-IV) for Korean adults. Research participants were selected based on a stratified multistage, cluster sampling design derived from a population census of community registry offices in 2010. The individual with the earliest birthday in the calendar year was selected from each included household. From the 7650 selected, 1628 refused (78.7% response rate), and 6022 subjects completed the face-to-face interviews [27]. Of these 6022 subjects, 1544 (25.6%) with a lifetime diagnosis of any DSM-IV psychiatric disorder were analyzed. The institutional review board of the Seoul National University College of Medicine approved the study protocol. Every subject was well-informed of the study objectives and methods prior to the interview. Written informed consent was obtained from each subject in advance of their participation in the study.

### Measurements

#### Mental health service utilization

Lifetime utilization of mental health service was determined by participant’s response to the following question: “Have you ever seen any professionals among the listed for mental health difficulties?” Professionals included in the list were psychiatrists; other type of mental health professionals encompassing psychologists, social workers, and nurses in a mental health specialty setting; non-psychiatric physicians; nurses in non-mental health specialty setting; acupuncturists; herbalists; pharmacists; and spiritual or religious advisors (e.g., shaman or priest). In this study, “mental health service” was defined to include mental health service by a psychiatrist, other type of mental health professional (e.g., psychologist, social worker, or nurse) in a mental health specialty setting, and non-psychiatric physician. Contact with other types of professionals was not regarded as mental health service in this study.

#### Personality characteristics

Personality traits were measured by Korean version of Big Five Inventory-10 (K-BFI-10) [28]. BFI-10 is a short-form of the Big-Five personality inventory that consists of two items identifying five personality traits, including neuroticism, openness, conscientiousness, agreeableness, and extraversion [29]. The reliability of BFI-K-10 Cronbach’s α = 0.52–0.75.

#### Psychiatric diagnoses

Psychiatric diagnoses were determined by Korean version of the Composite International Diagnostic Interview (K-CIDI). It is based on the Composite International Diagnostic Interview (CIDI) [30], a fully structured diagnostic modality to determine psychiatric diagnoses on the basis of the definitions and criteria of the DSM-IV [31]. The K-CIDI [32] was developed in accordance with WHO guidelines [33]. The inter-rater reliability, test/retest reliability, and validity of the K-CIDI range from 0.86–1.00, 0.42–0.89, and 0.50–1.00 kappa values, respectively.

#### Other variables

Self-reported socio-demographic data from the 2011 KECA included gender, age, family income, and marriage [27].

### Statistical analyses

Univariate logistic regression analyses was performed to compare between health service users and non-users concerning gender, age, economic status, and psychiatric diagnosis. Multivariate logistic regression analyses were performed to estimate the odds ratios (ORs) and 95% confidence intervals (CIs) of MHSU as the outcome variable and each personality construct score as the primary predictor. We examined two models to explore the effect of each personality construct on the MHSU. Model 1 included gender, age, and economic status as covariates. Model 2 additionally included the presence of alcohol use disorder, nicotine use disorder, depressive disorder, psychotic disorder, anxiety disorder, dysthymia, and somatoform disorder. Statistical analyses were done using SPSS version 21.0 (SPSS Inc., Chicago, IL) with significance level at *p*-value less than 0.05.

## Results

Of the 1544 participants with lifetime psychiatric disorders, 17.8% (*n* = 225; 95% CI = 15.9%–19.7%) had used mental health services. To identify possible confounders that may mediate the association between MHSU and personality constructs, we compared the socio-demographic characteristics and lifetime prevalence of psychiatric disorders between mental health service users and non-users. Mental health service users were older and more likely to be female and have low economic status compared to non-users. Depressive disorder (OR = 7.24, 95% CI = 5.45–9.62), dysthymia (OR = 3.41, 95% CI = 1.95–5.97), and anxiety disorder (OR = 2.15, 95% CI = 1.65–2.81) were positively associated with MHSU, and nicotine use disorder (OR = 0.54, 95% CI = 0.38–0.77) and alcohol use disorder (OR = 0.33, 95% CI = 0.24–0.45) were negatively associated with MHSU (Table 1).

**Table 1:** Socio-demographic and clinical correlates of mental health service utilization among KECA participants with lifetime mental disorder (*n* = 1544)

Independent variables	Users (*n* = 275)	Non-users (*n* = 1269)	Odd ratios (95% CI)	*P*-value
Female gender, n (%)	203 (73.8)	624 (49.2)	2.92 (2.18–3.90)	<0.001
Age,				
18–34	51 (18.5)	341 (26.9)	ref	
35–54	114 (41.5)	553 (43.6)	1.38 (0.97–1.97)	0.078
≥ 55	110 (40.0)	375 (29.6)	1.96 (1.36–2.82)	<0.001
Family income (per year)				
< 22,000 US	127 (59.9)	452 (42.9)	2.01 (1.40–2.89)	<0.001
22,000–33,000 US	38 (17.9)	265 (25.2)	1.03 (0.65–1.62)	0.915
> 33,000 US	47 (22.2)	336 (31.9)	ref	
Diagnosis, *n* (%)				
Nicotine use disorder	41 (14.9)	310 (24.5)	0.54 (0.38–0.77)	0.001
Alcohol use disorder	61 (22.3)	586 (46.4)	0.33 (0.24–0.45)	<0.001
Anxiety disorder	137 (51.3)	412 (32.9)	2.15 (1.65–2.81)	<0.001
Depressive disorder	175 (64.6)	225 (20.1)	7.24 (5.45–9.62)	<0.001
Dysthymic disorder	21 (9.3)	36 (2.9)	3.41 (1.95–5.97)	<0.001
Psychotic disorder	9 (3.3)	25 (2.0)	1.69 (0.78–3.66)	0.186
Somatoform disorder	17 (6.6)	85 (6.9)	0.96 (0.56–1.65)	0.884

Table 2 summarizes the associations between personality constructs and MHSU. After adjusting for age, gender, and economic status (Model 1), neuroticism (adjusted OR [AOR] = 1.30, 95% CI = 1.20–1.41) was positively associated with MHSU, and extraversion (AOR = 0.92, 95% CI = 0.86–0.99) and agreeableness (AOR = 0.89, 95% CI = 0.82–0.97) were negatively associated with MHSU. After further adjustment for DSM-IV psychiatric disorder in addition to age, gender, and economic status (Model 2), both neuroticism (AOR = 1.22, 95% CI = 1.10–1.34) and openness (AOR = 1.10, 95% CI = 1.00–1.22) were positively, and agreeableness (AOR = 0.89, 95% CI = 0.80–0.99) was negatively associated with MHSU.

**Table 2:** Multivariate analyses of personality variables and mental health utilization

Personality variables	Model 1		Model 2	
	AOR^a^ (95% CI)	*P*-value	AOR^b^ (95% CI)	*P*-value
Extraversion	0.92 (0.86–0.99)	0.023	0.98 (0.90–1.06)	0.976
Agreeableness	0.89 (0.82–0.97)	0.009	0.89 (0.80–0.99)	0.038
Conscientiousness	0.98 (0.90–1.06)	0.615	1.04 (0.94–1.15)	0.437
Neuroticism	1.30 (1.20–1.41)	<0.001	1.22 (1.10–1.34)	<0.001
Openness	1.06 (0.98–1.15)	0.140	1.10 (1.00–1.22)	0.048

^a^OR adjusted for age, sex, and family income

^b^OR adjusted for DSM-IV psychiatric disorders in addition to age, sex, and family income

## Discussion

In the KECA data, only 17.8% of patients with mental disorders reported ever using mental health services. Low use of mental health services among Koreans may be due to lack of mental health literacy and self-will to deal with mental illness by themselves [34]. Our results indicate that personality traits are closely associated with mental service utilization. Neuroticism, openness, and agreeableness were significantly associated with MHSU, after controlling for demographic and psychiatric features.

When comparing mental health service users and non-users, female gender, older age, and low income were related to the increased MHSU, consistent with prior findings from other national samples [2]. With regard to MHSU by psychiatric diagnoses, patients with nicotine use disorder and alcohol use disorder were less likely to seek help, while patients with internalizing disorders, including anxiety, depressive, and dysthymic disorder, were more likely to seek help. This is compatible with prior findings that only a few, 2.4% of, Chinese people with lifetime alcohol dependence sought professional treatment in Chinese [35]. According to the self-medication hypothesis, patients with substance use disorder often suffer with their emotional distress and tend to use substances to relieve and control their painful feelings [36]. Therefore, in our data, those with substance use disorders might have depended more on self-medication practices than on professional treatment for their emotional difficulties. Particularly, Koreans may not consider alcohol or nicotine use disorder as psychiatric problem, and therefore, may be unwilling to seek professional help, as found among Chinese population [35]. Accordingly, patients who self-medicate with substance for emotional distress may be less willing to seek and receive professional service for both their emotional and behavioral symptoms.

The association between neuroticism and MHSU found in our data has already been established in prior studies [9, 15, 37]. In previous results, neuroticism was related to MHSU [9] and increased number of mental health visits [15] in general population. In addition, among people with a subclinical depression, a greater level of neuroticism was associated with a greater tendency to perceive need for psychological care [37]. Given that neuroticism predicts more severe and impaired depressive symptoms [13], people who are high on neuroticism may actually experience more distressing symptoms which lead to greater professional help seeking. As neuroticism is associated with the lower problem-solving abilities [38] and difficulty in coping with negative emotions [39], it is also possible that people with high neuroticism may be less confident that they are able to solve their problems on their own by mobilizing their own supports. For instance, a neurotic patient may experience more difficulties handling his or her psychiatric symptoms, feeling overwhelmed, and therefore tend to be more dependent on professionals’ help.

In our data, openness to experience was significantly related to MHSU after controlling for psychiatric disorders. The results are inconsistent with the results of prior research failing to identify a significant relationship between openness and MHSU [9, 16]. However, a recent study found that openness was associated with increased active coping style and decreased avoidance to problems, and was also related to mental health care consumption for early psychotic symptoms [17]. Also, college students with lower openness to emotional experience and expression reported more negative attitudes toward seeking help for mental problems [40]. Taken together, openness may help a person to be less resistant to seek psychological help, particularly in Korea, where people are often pressured to inhibit their feelings and avoid shame related to mental illness [41].

An inverse association between agreeableness and MHSU was found in our data. There might be several possible explanations of the inverse association found in our data. One possibility is that the actual need for professional help might be lower among patients with high agreeableness than those with low agreeableness. For instance, patients with high agreeableness seem to have better social support [17, 42] and thus might be able to cope better with emotional difficulties without seeking professional help. Given the relevance of agreeableness to altruistic and sympathetic attitude and desire of giving help to help others [43–45], an alternative hypothesis may be that highly agreeable individuals focus less on their own difficulties than on those of others, and so will not actively seek for treatment despite their real need for professional help. Scholte-Stalenhoef et al. [17] found that individuals with high agreeableness were less likely to express their emotions, which was related to a decreased active coping style. Lastly, individuals with high agreeableness might be more sensitive to being negatively evaluated by others and have concerns for how others would view their mental difficulties or use of mental health service. For instance, agreeableness was found to be significantly correlated with the tendency to tailor one’s response to impress others (i.e., impression management), which might be related to the desire to preserve positive relations with others [46]. Considering the negative stigma toward mental illness among Asians [19], those with agreeableness traits might be more inhibited to present their difficulty to others. However, the data from this study cannot offer a clear account of this mechanism.

This study has several limitations. The cross-sectional design prevented investigation of the causal relationship between personality traits and MHSU. Second, we used lifetime MHSU rather than MHSU during a shorter period of time (e.g., prior 12 months). This may have lead to underestimation of MHSU due to recall bias over a longer duration. Finally, we only included community-dwelling individuals and not patients in institutional settings, which may have lead to the under-reporting of MHSU.

## Conclusions

The present study identified that MHSU in Korean population with mental disorder was affected by personality traits. The positive association of MHSU with neuroticism and openness and negative association with agreeableness were found in our data. Personality and individual different characteristics should be considered when implementing strategies for improving the access to professional help for those with mental health problems. Further research is needed to identify whether personality traits have a role in the resilience process or as determinants of MHSU.

## Abbreviations

CIDI: Composite International Diagnostic Interview
CIs: **C**onfidence intervals
DSM-IV: Diagnostic and statistical manual of mental disorders, Fourth Edition
K-BFI-10: Korean version of big five inventory-10
K-CIDI: Korean version of the composite international diagnostic interview
KECA: Korean epidemiologic catchment area
MHSU: Mental health service utilization
ORs: **O**dds ratios
